# Chemistry and Bioactivity of Briaranes from the South China Sea Gorgonian *Dichotella gemmacea*

**DOI:** 10.3390/md14110201

**Published:** 2016-10-28

**Authors:** Cui Li, Ming-Ping La, Hua Tang, Peng Sun, Bao-Shu Liu, Chun-Lin Zhuang, Yang-Hua Yi, Wen Zhang

**Affiliations:** Research Center for Marine Drugs, School of Pharmacy, Second Military Medical University, 325 Guo-He Road, Shanghai 200433, China; licuiwan@163.com (C.L.); lmp12@163.com (M.-P.L.); tanghua0309@126.com (H.T.); sunpeng78@126.com (P.S.); liubaoshu@126.com (B.-S.L.); zclnathan@163.com (C-L.Z.); yiyanghua@126.com (Y.-H.Y.)

**Keywords:** briarane diterpenoids, biological activity, structure elucidation, gorgonian, *Dichotella gemmacea*

## Abstract

Seven new briarane diterpenoids, gemmacolides AZ–BF (**1**–**7**), were isolated together with eight known analogues (**8**–**15**) from the South China gorgonian *Dichotella gemmacea*. Their structures were elucidated based on detailed spectroscopic analysis and a comparison with reported data. In an in vitro bioassay, these compounds exhibited different levels of growth inhibition activity against A549 and MG63 cells, giving continuous evidences about the biological contribution of functional groups at C-2, C-12, C-13, and C-16. These compounds were also evaluated for their antibacterial and antifungal activities. Compound **8** exhibited a potential antibacterial activity against both Gram-positive bacterium *Bacillus megaterium* and Gram-negative bacterium *Escherichia coli*.

## 1. Introduction

**Chart 1:**
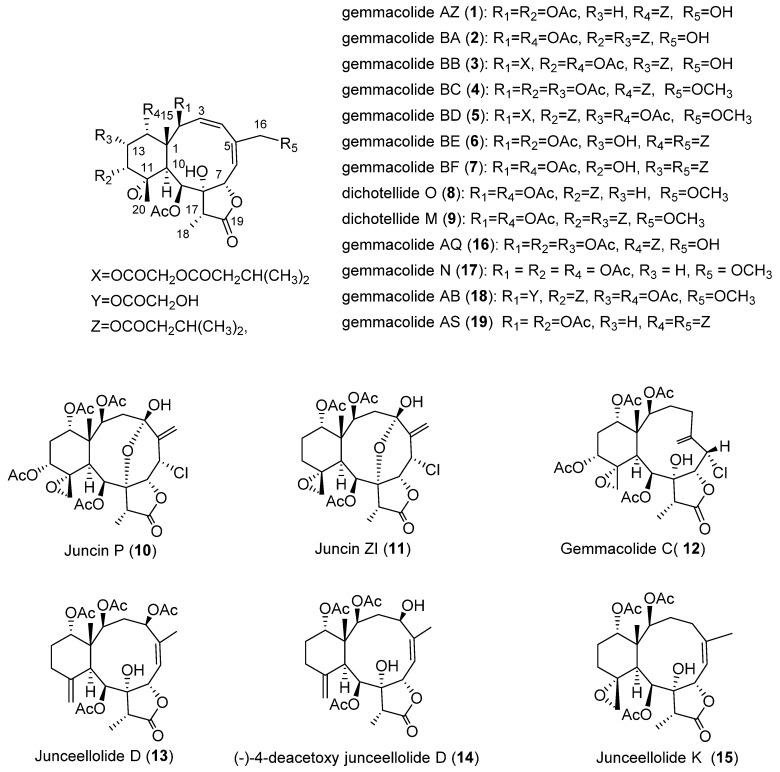
Structures of compounds **1**–**19**.

Briarane diterpenoids are a family of structurally intriguing metabolites that are mainly obtained from gorgonians [1]. These metabolites are reported to display a wide spectrum of bioactivities, including cytotoxic, anti-inflammatory, antiviral, antifouling, insecticidal, and immunomodulatory effects [1,2,3,4]. In the course of our searching for novel and bioactive secondary metabolites from the South China Sea invertebrates [5,6,7,8,9,10,11,12], the cluster of metabolites attracted our attention due to their significant tumor tell growth inhibitory activity [7,8,9,10,11,12]. Considering the structural complexity of the metabolites, specific research regarding the determination of absolute configuration was carried out using a solution TDDEFT/ECD approach [12]. Successive investigation on several collections of the gorgonian *Dichotella gemmacea* and *Junceella gemmacea* led to the isolation and structure elucidation of 48 new briaranes and 29 known analogues. On the basis of in vitro screening, a structure–activity relationship analysis of the metabolites was performed [7,8,9,10,11,12]. Three new briaranes, gemmacolides J, V, and Y, showed significant growth inhibitory activity towards A549 cells, being more active than the positive control adriamycin [10,11]. The initial research encourages continuous investigations of this class of metabolites, leading to the isolation and structure elucidation of seven new briaranes, namely gemmacolides AZ–BF (**1**–**7**), together with eight known analogues (**8**–**15**) [13,14,15,16,17,18,19] (Chart 1) from the gorgonian *D. gemmacea*. The structures of these compounds were elucidated on the basis of detailed spectroscopic analysis (Figures S1–S47) and a comparison with reported data. The isolates were tested in vitro for their antimicrobial and tumor cell growth inhibition activities. We herein report on the isolation, structure elucidation, and bioactivities of these compounds.

## 2. Results

Freshly collected specimens of *D. gemmacea* were immediately frozen to −20 °C and stored at this temperature before extraction. The usual workup for the extraction and isolation of briarane diterpenoids [8,9,10,11,12] yielded 15 pure compounds (**1**–**15**). The known compounds dichotelllides O and M (**8**, **9**) were once reported from the gorgonian *D. gemmacea* [13], while gemmacolide C (**12**) [18] was previously isolated from the gorgonians *J. gemmacea*. Juncins P (**10**) [14] and ZI (**11**) [16] were firstly isolated from the gorgonian *J. juncea*, and then repeatedly obtained from the gorgonians *D. gemmacea* [13,20,21], *D. fragilis* [22], *J. gemmacea* [7], and *J. fragilis* [23]. Junceellolide D (**13**) [17], (−)-4-deacetoxy junceellolide D (**14**) [15], and junceellolide K (**15**) [19] were firstly isolated from the gorgonians *J. fragilis* and then re-isolated from many gorgonian corals, including *Verrucella umbraculum* [24], *J. fragilis* [23,25,26,27], *D. gemmacea* [8,13,20], *J. gemmacea* [7], *Menella* sp. [28], and *J. juncea* [14]. These metabolites displayed antifouling, anti-inflammatory, and cytotoxic activities in the in vitro bioscreening [13,16,21,22,29].

Gemmacolide AZ (**1**) was isolated as a white amorphous powder. The molecular formula C_31_H_42_O_13_ was established by the HRESIMS. The IR spectrum showed strong absorption bands of hydroxyl (3468 cm^−^^1^), γ-lactone (1775 cm^−^^1^), and ester (1738 cm^−^^1^) functionalities. This observation was in agreement with the signals in the ^13^C NMR and DEPT spectra (Table 1) for 9 *sp*^2^ carbon atoms (5 × OC = O, CH = CH, CH = C) at lower field and 22 *sp*^3^ carbon atoms at higher field (1 × C, 3 × CH, 2 × CH_2_, 7 × CH_3_, 2 × OC, 5 × OCH, 2 × OCH_2_), accounting for seven double bond equivalents. The remaining double bond equivalents were due to the presence of four rings in the molecule.

**Figure 1:**
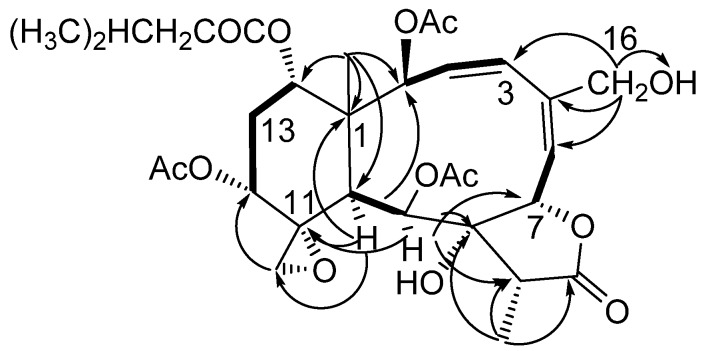
Key HMBC (arrow H → C) and COSY (bond) spin coupling systems for compound **1**.

**Figure 2:**
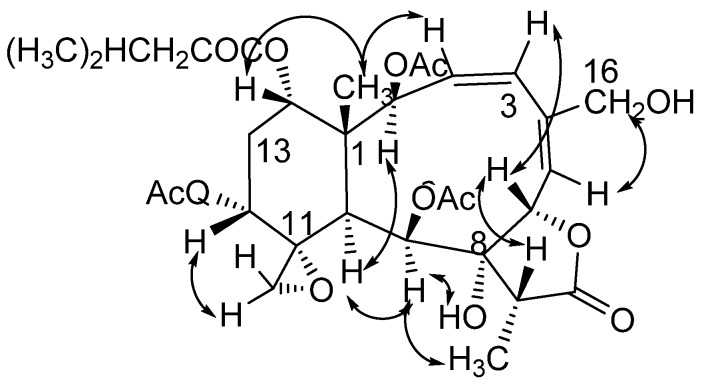
Key NOESY correlations for compound **1**.

The ^1^H and ^13^C NMR spectra data (Table 1 and Table 2) of **1** showed great similarity to those of gemmacolide AQ (**16**) [9], except for the absence of an acetoxy group. The missing group was readily assigned at C-13 due to the proton sequence of H-12/H_2_-13/H-14, established by ^1^H–^1^H COSY experiment (Figure 1). The assignment was fully supported by the ^1^H–^1^H COSY and HMBC spectra as shown in Figure 1. The relative configuration of **1** at chiral centers was proven the same as that of **16** by a NOESY experiment (Figure 2), showing a β configuration of H-7, H-12, H-14, Me-15, H-17, and CH_2_-20, and an α configuration of H-2, H-9, H-10, and Me-18. The geometry of the Δ^3^ double bond was assigned as (*Z*) based on the proton coupling constant between H-3 and H-4 (*J* = 10.6 Hz), while Δ^5,6^ was determined as (*E*) due to the NOESY correlation between H-6 and H_2_-16. The NMR assignments of **1** were further supported by a comparison with the reported data of gemmacolide N (**17**), an analogue obtained from the same species of animals with its absolute stereochemistry being determined by a detailed TDDFT/ECD analysis [12]. Compound **17** differs from **1** in the functional groups at C-14 and C-16, having acetoxy and primary hydroxyl groups instead of isovaleryl and methoxy groups, respectively. As compound **1** contained the same lactone and diene chromophores as gemmacolide N [12], and they differed only in the nature of the ester groups and R_5_, the ECD spectrum of gemmacolide N could be used as ECD reference for the configurational assignment. The absolute configuration of **1** is therefore determined as (1*R*,2*S*,7*S*,8*S*,9*S*,10*S*,11*R*,12*R*,14*S*,17R).

Gemmacolide BA (**2**), a white amorphous powder, had the molecular formula of C_36_H_50_O_15_ on the basis of its HRESIMS. Its ^1^H and ^13^C NMR spectra data (Table 1 and Table 2) were similar to those of gemmacolide AQ (**16**) [9]. However, an acetyl group in **16** was replaced by an isovaleryl group in **2**. The three acetyl groups were assigned at C-2, C-9, and C-14 due to the obvious HMBC correlations from the secondary alcohol protons to the respective ester carbonyl groups. The location of two isovaleryl groups at C-12 and C-13 was supported by the obvious HMBC correlations from H-12 and H-13 to the respective carbonyl carbon of the isovaleryl groups. The relative configuration and the absolute configuration of **2** were proven to be the same as that of **16** on the basis of NOESY experiment and their similar ECD spectra.

Gemmacolide BB (**3**) was obtained as a white amorphous powder with the molecular formula of C_38_H_52_O_17_ being established by HRESIMS. The ^1^H and ^13^C NMR spectra of **3** showed similarity to those of compound **2** (Table 1 and Table 2). The structure of **3** differed from that of **2** with the presence of an isovaleric acetyl group instead of an acetyl group at C-2 and an acetyl group replace of the isovaleryl group at C-12 (Table 1 and Table 2). The conclusion was fully supported by the distinct HMBC correlations from H-2 to the carbonyl carbons of the isovaleric acetyl group at δ 167.8, and from H-12 to the acetyl ester carbon at δ 169.8, respectively. Its absolute configuration was proven to be the same as that of **2** on the basis of their similar ECD spectra.

Gemmacolide BC (**4**) was obtained as a white amorphous powder. Its HRESIMS demonstrated the same molecular formula as C_34_H_46_O_15_ by HRESIMS. Comparison of overall ^1^H and ^13^C NMR spectra data (Table 1 and Table 2) of **4** with those of gemmacolide AQ (**16**) [9] revealed great similarity with the only difference of an appearance of an oxygenated methyl group at C-16 (δ_H_ 3.44, s; δ_C_ 58.5, CH_3_). The result was supported by the obvious HMBC correlations from the methoxyl protons to C-16. The absolute structure of **4** was proven to be the same as that of **16** by the analysis of NOESY and ECD spectra. 

Gemmacolide BD (**5**) was obtained as a white amorphous powder and exhibited a molecular formula of C_39_H_54_O_17_ as deduced from its HRESIMS. ^1^H and ^13^C NMR spectra of **5** (Table 1 and Table 3) were very similar to those of compound gemmacolide AB (**18**) [9]. The primary alcohol of the hydroxyacetyl group, however, was further isovalerianated. This conclusion was proved by the long-range correlation from secondary hydrogens of both the isovaleryl group and the hydroxyacetyl group to the carbonyl carbon of the isovaleryl group (δ 172.3), and from both H-2 and the secondary hydrogens of the hydroxyacetyl group to the carbonyl carbon of the hydroxyacetyl group (δ 166.6). The same absolute geometry was obtained on the basis of the ECD spectrum.

Gemmacolide BE (**6**) was found to be a white amorphous powder, having the molecular formula of C_36_H_50_O_15_ based on the HRESIMS. ^1^H and ^13^C NMR spectra (Table 1 and Table 3) of **6** resembled to those of gemmacolide AS (**19**) [8] except for a hydrogen at C-13 in **19** was replaced by a hydroxyl group in **6**. The assignment was in agreement with its NMR shift values (δ_H-13_ 4.07, br s; δ_C-13_ 67.4, CH), compared to those of the ester group substitution in gemmacolide P, gemmacolide AC, gemmacolide AD, gemmacolide AF, gemmacolide AG, gemmacolide AR, and gemmacolide AT (δ_H-13_ 5.08–5.10, d, *J* = 2.7–3.5; δ_C-13_ 66.3–66.7, CH) [8,9,12], and further supported by the proton sequences from H-12 to H-14 established by the ^1^H–^1^H COSY experiment. The hydroxyl group was assigned as an α-orientation due to the NOESY correlation of H-13 with H-15. The two isovaleryl groups were deduced to be attached to C-14 and C-16 based on the 2D NMR (^1^H–^1^H COSY, HMBC) analysis and a comparison to those reported data of analogues [7,8,9,10,11,12]. The relative and absolute configuration of **6** was also proven to be the same as those of **19** by the NOESY and ECD experiments.

Gemmacolide BF (**7**) was a white amorphous powder and had the same molecular formula of C_36_H_50_O_15_ as that of **6** as deduced from its HRESIMS. The structure of **7** differed from that of **6** only in the sequence of substituent groups. The hydroxyl, isovaleryl, and acetoxy groups at C-13, C-14, and C-12 in **6** were instead assigned at C-12, C-13, and C-14 in **7**, respectively. The location of hydroxyl at C-12 was supported by ^1^H and ^13^C NMR spectra data (δ_H-12_ 3.48, br d, *J* = 4.7; δ_C-12_ 75.3, CH), compared to those of ester group substitution (δ_H-12_ 4.88–4.93, br d, *J* = 2.8–3.5; δ_C-12_ 72.8–73.3, CH) [8,9,12]. A β-orientation of H-12 was deduced from its NOESY correlation with H-20b. Two isovaleryl groups were attached at C-13 and C-16 due to the HMBC correlations of H-13 and H-16 with the respective carbonyl carbon of the isovaleryl groups. The assignment was supported by the proton sequence of H-12/H-13/H-14, as deduced from the ^1^H–^1^H COSY experiment. The established structure of **7** was further supported by a detailed analysis of its 1D NMR and 2D NMR data. Its absolute configuration was determined as (−)-(1*S*,2*S*,3*Z*,5*E*,7*S*,8*S*,9*S*,10*S*,11*R*,12*R*,13*S*,14*R*,17*R*) based on NOESY and ECD experiments.

The compounds were evaluated for their antimicrobial and tumor cell growth inhibition activities. In the in vitro bioassays, compounds **1**–**9** exhibited a different level of growth inhibition against cell lines A549 and MG63 (Table 4). The activity of **5** with 2-isovaleric acetyl substitution marked decreased compared with its 2-glycolyl analogue (IC_50_ for A549 = 19.4 μM, for MG63 = 22.8 μM) [9]. The previous conclusion that 13-isovalerate may decrease the activity [9] was further supported by comparing the activity of **1** and **9** with those of **16** (IC_50_ = 28.7 and > 100 μM for A549 and MG63, respectively) [9] and **8**, respectively. Comparing the activity of **2** and **8** with respect to those of gemmacolides AT (IC_50_ > 30.0 μM for both A549 and MG63) [8] and N (IC_50_ > 50.5 μM for both A549 and MG63) [12] supported the conclusion that 12-*O*-isovalerate gave a positive contribution to the activity as mentioned previously [9,11,12]. The replacement of an isovaleryl group by an acetyl group at C-14 would increase the activity as observed in **4** and juncenolide D [12]. The 16-substituents displayed an activity order as 16-OH > 16-OMe when the activity of **2** and **3** was compared with those of **9** (IC_50_ > 30.0 μM for both A549 and MG63) and gemmacolide AL (IC_50_ > 37.8 μM for both A549 and MG63) [9], respectively.

In antimicrobial test in vitro, compounds **4** and **8** showed potential antibacterial activity against the Gram-negative bacterium *Escherichia coli*, while **8** exhibited significant antibacterial activity against the Gram-positive bacterium *Bacillus megaterium*. Compounds **1**, **2**, **4**, **5**, and **8** exhibited weak antifungal activity against both *Septoria tritici* and *Microbotryum violaceum*, while compound **9** only displayed weak activity against *S. tritici* (Table 5).

## 3. Materials and Methods

### 3.1. General Experimental Procedures

Commercial silica gel (Yantai, China, 200–300; 400–500 mesh) and RP silica gel (Merck, Darmstadt, Germany, 43–60 μm) were used for column chromatography (CC). Precoated silica gel plates (Yantai, China, HSGF-254) and RP silica gel (Macherey-Nagel, Düren, Germany, RP-18 F254) were used for analytical thin-layer chromatography (TLC). Spots were detected on TLC under UV or by heating after spraying with an anisaldehyde-sulphuric acid reagent. The NMR spectra were recorded at 300 K on a Bruker DRX 400 spectrometer (Ettlingen, Germany). Chemical shifts are reported in parts per million (δ), with use of the residual CHCl_3_ signal (δ_H_ 7.26 ppm) as an internal standard for ^1^H NMR and CDCl_3_ (δ_C_ 77.0 ppm) for ^13^C NMR; Coupling constants (*J*) are reported in Hz. ^1^H NMR and ^13^C NMR assignments were complemented ^1^H–^1^H COSY, HSQC, HMBC, and NOESY experiments. The following abbreviations are used to describe spin multiplicity: s = singlet; d = doublet; t = triplet; q = quartet; m = multiplet; br s = broad singlet; dd = doublet of doublets; ov = overlapped signals. Optical rotations were measured in CHCl_3_ with an Autopol IV polarimeter at the sodium D line (590 nm). Infrared spectra were recorded in thin polymer films on a Nexus 470 FT-IR spectrophotometer (Nicolet, Madison, WI, USA); peaks are reported in cm^−1^. UV absorption spectra were recorded on a Varian Cary 100 UV-Vis spectrophotometer (Palo Alto, CA, USA); peaks wavelengths are reported in nm. Circular dichroism spectra were recorded with a JASCO J-715 circular dichroism spectropolarimeter (Mary’s Court Easton, MD, USA). The MS and HRMS were performed on a Q-TOF Micro mass spectrometer (Bruker, Bremen, Germany), resolution 5000. An isopropyl alcohol solution of sodium iodide (2 mg/mL) was used as a reference compound. Semi-preparative RP-HPLC was performed on an Agilent 1100 system (Santa Clara, CA, USA) equipped with a refractive index detector using an YMC Pack ODS-A column (Kyoto, Japan, particle size 5 μm, 250 mm × 10 mm).

### 3.2. Animal Material

The South China Sea gorgonian coral *Dichotella gemmacea* (3.5 kg, wet weight) was collected from the South China Sea in August 2007 and identified by Xiu-Bao Li, South China Sea Institute of Oceanology, Chinese Academy of Sciences. A voucher specimen (ZS-3) was deposited in the Second Military Medical University.

### 3.3. Extraction and Isolation

The frozen specimen was extracted ultrasonically three times with acetone and MeOH, respectively. The combined residue was partitioned between H_2_O and EtOAc to afford 16.1 g of an EtOAc extract. The EtOAc extract was further partitioned between MeOH and hexane, affording 11.2 g of MeOH soluble residue. The MeOH extract was subjected to column chromatography (CC) on silica gel to give 16 fractions, using hexane and acetone (from 100:0 to 0:100) as an eluent. Fraction 5 was repeatedly subjected to normal phase silica gel column chromatography, Sephadex LH-20, and RP-silical gel column chromatography, then purified via HPLC (eluent MeOH/H_2_O, 75:25, 1.5 mL/min), yielding **5** (3.4 mg) at 29.6 min. Fraction 6 was chromatographed over Sephadex LH-20 to give four sub-fractions (A–D) eluted with CHCl_3_ and MeOH (1:1). Sub-fractions A was purified via HPLC (eluent MeOH/H_2_O, 70:30, 1.5 mL/min), yielding **2** (2.0 mg) at 34.0 min. Sub-fractions B was purified via HPLC (eluent MeOH/H_2_O, 70:30, 1.5 mL/min), yielding **6** (1.4 mg) and **7** (1.6 mg) at 64.3 and 67.9 min. Sub-fractions C was purified via HPLC (eluent MeOH/H_2_O, 70:30, 1.5 mL/min), yielding **3** (1.0 mg) at 62.3 min. Fraction 10 was repeatedly chromatographed over normal phase silica gel column and Sephadex LH-20 and purified via HPLC (eluent MeOH/H_2_O, 70:30, 1.5 mL/min), yielding **4** (3.4 mg) at 51.7 min. Fraction 13 was further fractionated by RP-silical gel column chromatography (gradient elution from MeOH/H_2_O, 2:7 to 2:1, in 5% increments) to afford 6 sub-fractions (A–F), and sub-fraction E was purified via HPLC (eluent MeOH/H_2_O, 65:35, 1.5 mL/min), yielding **1** (2.3 mg) at 29.7 min.

Gemmacolide AZ (**1**): white amorphous powder; ${\left[\alpha \right]}_{D}^{24}$ −1.3 (*c* 0.07, CHCl_3_); UV (MeOH) λ_max_(log ε) 205 (1.71) nm; CD (CH_3_CN, *c* 2.0 × 10^−4^) *λ*_max_ (Δε) positive below 190 nm, 209 (−5.50) nm; IR (film) ν_max_ 3468, 1775, 1738 cm^−1^; ^1^H NMR spectroscopic data, see Table 2; ^13^C NMR spectroscopic data, see Table 1; ESIMS *m*/*z* 645 [M + Na]^+^; HRESIMS *m*/*z* 645.2527 [M + Na]^+^ (calcd. for C_31_H_42_O_13_ Na, 645.2523).

Gemmacolide BA (**2**): white amorphous powder; ${\left[\alpha \right]}_{D}^{24}$ −43.0 (*c* 0.07, CHCl_3_); UV (MeOH) λ_max_(log ε) 208 (2.26) nm; CD (CH_3_CN, *c* 4.2 × 10^−4^) λ_max_ (Δε) positive below 190 nm, 209 (−4.77) nm; IR (film) ν_max_ 3469, 1775, 1744 cm^−^^1^; ^1^H NMR spectroscopic data, see Table 2; ^13^C NMR spectroscopic data, see Table 1; ESIMS *m*/*z* 745 [M + Na]^+^; HRESIMS *m*/*z* 745.3041 [M + Na]^+^ (calcd. for C_36_H_50_O_15_Na, 745.3047).

Gemmacolide BB (**3**): white amorphous powder; ${\left[\alpha \right]}_{D}^{24}$ −28 (*c* 0.03, CHCl_3_); UV (MeOH) λ_max_(log ε) 204(1.47) nm; CD (CH_3_CN, *c* 1.6 × 10^−4^) λ_max_ (Δε) positive below 190 nm, 205 (−5.36) nm; IR (film) ν_max_ 3469, 1772, 1744 cm^−^^1^; ^1^H NMR spectroscopic data, see Table 2; ^13^C NMR spectroscopic data, see Table 1; ESIMS *m*/*z* 803 [M + Na]^+^; HRESIMS *m*/*z* 803.3107 [M + Na]^+^ (calcd. for C_38_H_52_O_17_Na, 803.3102).

Gemmacolide BC (**4**): white amorphous powder; ${\left[\alpha \right]}_{D}^{24}$ −44.0 (*c* 0.24, CHCl_3_); UV (MeOH) λ_max_(log ε) 205 (1.55) nm; CD (CH_3_CN, *c* 4.1 × 10^−4^) λ_max_ (Δε) positive below 190 nm, 201.5 (−4.62) nm; IR (film) ν_max_ 3479, 1776, 1743 cm^−^^1^; ^1^H NMR spectroscopic data, see Table 2; ^13^C NMR spectroscopic data, see Table 1; ESIMS *m*/*z* 717 [M + Na]^+^; HRESIMS *m*/*z* 717.2730 [M + Na]^+^ (calcd. for C_34_H_46_O_15_Na, 717.2734).

Gemmacolide BD (**5**): white amorphous powder; ${\left[\alpha \right]}_{D}^{24}$ −36.0 (*c* 0.12, CHCl_3_); UV (MeOH) λ_max_(log ε) 205 (1.61) nm; CD (CH_3_CN, *c* 2.0 × 10^−4^) λ_max_ (Δε) positive below 190 nm, 216 (−7.34) nm; IR (film) ν_max_ 3461, 1775, 1744 cm^−^^1^; ^1^H NMR spectroscopic data, see Table 3; ^13^C NMR spectroscopic data, see Table 1; ESIMS *m*/*z* 817 [M + Na]^+^; HRESIMS *m*/*z* 817.3254 [M + Na]^+^ (calcd. for C_39_H_54_O_17_Na, 817.3259).

Gemmacolide BE (**6**): white amorphous powder; ${\left[\alpha \right]}_{D}^{24}$ −22.0 (*c* 0.05, CHCl_3_); UV (MeOH) λ_max_(log ε) 204 (1.41) nm; CD (CH_3_CN, *c* 3.5 × 10^−4^) λ_max_ (Δε) positive below 190 nm, 198.0 (−5.43) nm; IR (film) ν_max_ 3467, 1776, 1741 cm^−^^1^; ^1^H NMR spectroscopic data, see Table 3; ^13^C NMR spectroscopic data, see Table 1; ESIMS *m*/*z* 745 [M + Na]^+^; HRESIMS *m*/*z* 745.3043 [M + Na]^+^ (calcd. for C_36_H_50_O_15_Na, 745.3047).

Gemmacolide BF (**7**): white amorphous powder; ${\left[\alpha \right]}_{D}^{24}$ −30.0 (*c* 0.05, CHCl_3_); UV (MeOH) λ_max_(log ε) 204 (1.53) nm; CD (CH_3_CN, *c* 1.7 × 10^−4^) λ_max_ (Δε) positive below 190 nm, 198.0 (−10.59) nm; IR (film) ν_max_ 3469, 1777, 1742 cm^−^^1^; ^1^H NMR spectroscopic data, see Table 3; ^13^C NMR spectroscopic data, see Table 1; ESIMS *m*/*z* 745 [M + Na]^+^; HRESIMS *m*/*z* 745.3051 [M + Na]^+^ (calcd. for C_36_H_50_O_15_Na, 745.3051).

### 3.4. Cytotoxicity Assay

Cytotoxicity was tested against human lung adenocarcinoma (A549) and human osteosarcoma cell (MG63), using a modification of the MTT [3-(4,5-dimethylthiazol-2-yl)-2,5-diphenyltetrazolium bromide] colorimetric method [30]. DMSO was used as a vehicle control, and adriamycin was used as a positive control, IC_50_ = 2.8 μM for A549 cells and 3.2 μM for MG63 cells.

### 3.5. Agar Diffusion Test for Biological Activity

Compounds **1**–**9** were dissolved in acetone at a concentration of 2 mg/mL; 25 μL of the solution (0.05 mg) were pipetted onto a sterile filter disc (Schleicher & Schuell, Dassel, Germany, 9 cm), which was placed onto an appropriate agar growth medium for the respective test organism and subsequently sprayed with a suspension of the test organize. The test organisms were the Gram-negative bacterium *Escherichia coli* (Coli), the Gram-positive bacterium *Bacillus megaterium* (Meg) (both grown on NB medium), the fungus *Microbotryum violaceum* (Vio), and *Septoria tritici* (Tri) (both grown on MPY medium). Commencing at the outer edge of the filter disc, the diameter of zone of inhibition was measured in mm. Reference substances were ketoconazole (0.05 mg), penicillin (0.05 mg), and streptomycin (0.05 mg), with the diameter (Ф) of zone of inhibition being 18.0, 18.0, 30.0, 25.0; 26.0, 18.0, 14.0, 12.0; and 18.0, 11.0, 16.0, 11.0 mm, respectively.

## Figures and Tables

**Table 1:** ^13^C NMR data for gemmacolides AZ–BF (**1**–**7**) ^a^.

Position	1	2	3	4	5	6	7
1	47.2, C	46.5, C	46.5, C	46.5, C	46.5, C	46.3, C	46.5, C
2	75.8, CH	75.6, CH	76.9, CH	74.2, CH	75.4, CH	74.4, CH	74.1, CH
3	131.5, CH	131.2, CH	130.5, CH	131.3, CH	130.5, CH	131.2, CH	132.2, CH
4	129.2, CH	129.6, CH	130.2, CH	128.6, CH	129.4, CH	127.8, CH	127.7, CH
5	144.7, C	144.5, C	144.6, C	141.7, C	141.5, C	138.9, C	139.0, C
6	123.4, CH	123.7, CH	124.4, CH	122.7, CH	122.6, CH	122.4, CH	122.3, CH
7	78.9, CH	78.7, CH	78.7, CH	79.0, CH	79.0, CH	78.7, CH	78.5, CH
8	81.2, C	81.0, C	81.1, C	81.1, C	81.0, C	81.0, C	81.1, C
9	64.1, CH	63.8, CH	63.8, CH	63.8, CH	63.9, CH	63.9, CH	64.0, CH
10	32.9, CH	32.8, CH	32.6, CH	32.7, CH	32.7, CH	32.7, CH	31.5, CH
11	59.2, C	58.4, C	58.3, C	58.4, C	58.2, C	58.7, C	60.4, C
12	73.0, CH	72.8, CH	73.1, CH	73.3, CH	72.8, CH	76.5, CH	75.3, CH
13	29.0, CH_2_	66.2, CH	66.2, CH	66.5, CH	66.4, CH	67.4, CH	67.3, CH
14	73.2, CH	73.8, CH	73.8, CH	73.4, CH	73.8, CH	76.6, CH	76.1, CH
15	14.2, CH_3_	14.5, CH_3_	14.4, CH_3_	14.5, CH_3_	14.4, CH_3_	14.8, CH_3_	14.6, CH_3_
16	63.9, CH_2_	63.9, CH_2_	63.9, CH_2_	72.1, CH_2_	71.9, CH_2_	62.8, CH_2_	62.8, CH_2_
17	44.2, CH	44.1, CH	44.1, CH	44.2, CH	44.2, CH	44.1, CH	44.0, CH
18	6.4, CH_3_	6.3, CH_3_	6.3, CH_3_	6.3, CH_3_	6.3, CH_3_	6.3, CH_3_	6.4, CH_3_
19	175.4, C	175.3, C	175.7, C	175.3, C	175.3, C	175.3, C	175.4, C
20	49.1, CH_2_	49.1, CH_2_	49.0, CH_2_	49.1, CH_2_	49.0, CH_2_	48.9, CH_2_	48.2, CH_2_
9-OAc	170.3, C	170.2, C	170.1, C	170.2, C	170.2, C	170.2, C	170.2, C
	21.6, CH_3_	21.6, CH_3_	21.5, CH_3_	21.5, CH_3_	21.5, CH_3_	21.5, CH_3_	21.6, CH_3_
R_1_	170.5, C	170.9, C	167.8, C	169.5, C	166.6, C	169.6, C	169.8, C
	21.3, CH_3_	21.6, CH_3_	61.0, CH_2_	20.6, CH_3_	60.8, CH_2_	21.3, CH_3_	21.3, CH_3_
			172.3, C		172.3, C		
			42.7, CH_2_		42.7, CH_2_		
			25.5, CH		25.6, CH		
			22.3, 2 × CH_3_		22.5, 2 × CH_3_		
R_2_	170.1, C	171.8, C	169.8, C	169.9, C	171.8, C	171.8, C	
	21.3, CH_3_	43.5, CH_2_	20.8, CH_3_	21.1, CH_3_	43.5, CH_2_	21.0, CH_3_	
		25.7, CH			25.7, CH		
		22.4, 2 × CH_3_			22.5, 2 × CH_3_		
R_3_		171.7, C	171.2, C	169.7, C	169.6, C		172.0, C
		42.6, CH_2_	42.6, CH_2_	21.3, CH_3_	20.5, CH_3_		42.8, CH_2_
		25.0, CH	25.0, CH				25.2, CH
		22.4, 2 × CH_3_	22.3, 2 × CH_3_				22.3, 2 × CH_3_
R_4_	172.0, C	170.1, C	170.1, C	172.3, C	170.2, C	173.7, C	169.8, C
	42.9, CH_2_	20.9, CH_3_	20.8 CH_3_	43.3, CH_2_	20.8, CH_3_	43.3, CH_2_	20.9, CH_3_
	24.8, CH			25.0, CH		25.6, CH	
	22.6; 22.4, CH_3_			22.5, 2 × CH_3_		22.4, 2 × CH_3_	
R_5_				58.5, CH_3_	58.5, CH_3_	172.1, C	172.0, C
						43.3, CH_2_	43.2, CH_2_
						25.7, CH	25.7, CH
						22.4, 2 × CH_3_	22.4, 2 × CH_3_

^a^ 100 MHz, in CDCl_3_, assignments made by DEPT, ^1^H–^1^H COSY, HSQC, and HMBC.

**Table 2:** ^1^H NMR data for gemmacolides AZ–BC (**1**–**4**) ^a^.

Position	1	2	3	4
2	5.68, d (9.5)	5.62, d (9.4)	5.71, d (9.3)	5.52, d (9.5)
3	5.60, dd (10.6, 9.5)	5.55, dd (9.5, 9.4)	5.63, dd (9.9, 9.3)	5.56, dd (9.8, 9.5)
4	6.33, d (10.6)	6.35, d (9.5)	6.39, d (9.9)	6.28, d (9.8)
6	5.82, d (8.5)	5.81, d (8.5)	5.84, d (8.6)	5.88, d (8.5)
7	4.98, ov	4.96, d (8.5)	4.95, d (8.6)	4.96, d (8.5)
9	4.80, br d (4.7)	4.74, br d (4.8)	4.74, br d (4.7)	4.74, br d (4.5)
10	3.66, br d (4.5)	3.60, br d (4.8)	3.62, ov	3.61, ov
12	4.54, br s	4.93, br d (2.3)	4.90, br d (2.4)	4.89, br s
13β	2.25, ov	5.11, t (3.4)	5.09, t (3.0)	5.10, br s
13α	1.96, ov			
14	4.99, br s	5.23, br d (2.4)	5.19, br d (2.2)	5.25, br s
15	1.06, s	1.14, s	1.13, s	1.13, s
16a	4.49, br s	4.47, br s	4.49, d (16.0)	4.49, d (14.9)
16b	4.49, br s		4.42, d (16.0)	4.22, d (14.9)
17	2.31, ov	2.30, ov	2.30, ov	2.29, ov
18	1.16, d (7.0)	1.13, d (7.2)	1.14, d (7.1)	1.15, ov
20a	3.55, br d (2.0)	3.62, br d (2.3)	3.62, br s	3.61, br s
20b	2.79, br d (2.0)	2.95, br d (2.3)	2.92, br s	2.92, br s
9-OAc	2.19, s	2.20, s	2.19, s	2.19, s
R_1_	1.98, s	1.99, s	4.55, d (15.7)	1.95, s
			4.44, d (15.7)	
			2.28, ov (×2)	
			2.13, ov	
			0.98, d (6.6) (×2)	
R_2_	2.12, s	2.32, m	2.16, s	2.16, s
		2.26, m		
		2.17, m		
		1.01, d (6.2)		
		0.99, d (6.2)		
R_3_		2.07, m (×2)	2.08, ov (×2)	1.95, s
		2.00, m	1.98, m	
		0.92, d (6.6) (×2)	0.92, d (6.5) (×2)	
R_4_	2.25, ov,	2.09, s	2.09, s	2.28, ov,
	2.07, ov			2.15, ov
	2.15, ov			2.11, ov
	0.97, d (6.3)			1.00, d (6.5)
	0.94, d (6.4)			0.97, d (6.5)
R_5_				3.44, s

^a^ 400 MHz, in CDCl_3_, assignments made by ^1^H–^1^H COSY, HSQC, HMBC, and NOESY.

**Table 3:** ^1^H NMR data for gemmacolides BD–BF (**5**–**7**) ^a^.

Position	5	6	7
2	5.64, d (9.6)	5.62, ov	5.66, ov
3	5.60, dd (10.3, 9.6)	5.61, ov	5.61, dd (10.4, 9.9)
4	6.33, d (10.3)	6.29, d (8.1)	6.28, d (10.4)
6	5.88, d (8.4)	5.71, d (8.3)	5.66, ov
7	4.98, d (8.4)	4.98, d (8.3)	4.98, ov
9	4.75, br d (4.5)	4.72, ov	4.78, br d (4.8)
10	3.62, br d (4.5)	3.59, br d (4.9)	3.56, br s
12	4.91, br d (2.2)	4.74, ov	3.48, br d (4.7)
13β	5.08, t (3.3)	4.07, br s	4.99, ov
13α			
14	5.18, br d (2.6)	5.22, br s	5.33, br s
15	1.13, s	1.09, s	1.13, s
16a	4.52, d (15.3)	5.43, d (16.0)	5.46, d (16.6)
16b	4.11, d (15.3)	4.64, d (15.4)	4.55, d (15.6)
17	2.30, ov	2.29, ov	2.28, ov
18	1.14, ov	1.14, d (7.1)	1.16, d (7.2)
20a	3.59, br d (2.1)	3.60, br d (2.2)	3.56, br s
20b	2.92, br d (2.1)	2.87, br d (2.2)	2.78, br s
9-OAc	2.18, s	2.18, s	2.19, s
R_1_	4.52, d (15.7)	1.98, s	1.94, s
	4.43, d (15.7)		
	2.29, ov (×2)		
	2.11, ov		
	0.98, ov (×2)		
R_2_	2.19, ov,	2.10, s	3.31, br d (4.7)
	2.33, ov		
	2.15, ov		
	0.98, ov		
	0.98, ov		
R_3_	1.94, s	3.01, br s	2.16, ov (×2)
			2.06, ov
			0.96, d (6.5)
			0.94, d (6.5)
R_4_	2.05, s	2.32, ov (×2)	2.13, s
		2.14, ov	
		0.99, ov (×2)	
R_5_	3.44, s	2.32, ov (×2)	2.30, ov (×2)
		2.14, ov	2.13, ov
		0.99, ov (×2)	0.99, ov (×2)

^a^ 400 MHz, in CDCl_3_, assignments made by ^1^H–^1^H COSY, HSQC, HMBC, and NOESY.

**Table 4:** Cytotoxic assay for compounds **1**–**9** (IC_50_ μM).

Compound	A549	MG63	Compound	A549	MG63
**1**	28.3	72.0	**6**	25.8	30.6
**2**	24.7	15.8	**7**	13.7	34.8
**3**	34.1	43.2	**8**	25.5	36.8
**4**	26.8	11.4	**9**	>40.8	>40.8
**5**	>37.8	>37.8	**Adriamycin**	2.8	3.2

**Table 5:** Agar diffusion assays for antibacterial and antifungal activities ^a,b^.

	*E. coli*	*B. megaterium*	*M. violaceum*	*S. tritici*
**1**	16.0	8.0	10.0	19.0
**2**	14.0	11.0	11.0	11.0
**3**	/	/	/	/
**4**	18.0	11.0	11.0	17.0
**5**	11.0	11.0	13.0	13.0
**6**	/	/	/	/
**7**	/	/	/	/
**8**	21.0	19.0	11.0	14.0
**9**	14.0	0	0	14.0
**ketoconazole**	18.0	18.0	30.0	25.0
**penicillin**	26.0	18.0	14.0	12.0
**streptomycin**	18.0	11.0	16.0	11.0

^a^ 0.05 mg of the test or control substances dissolved in acetone were applied to a filter disc and sprayed with the respective test organism; ^b^ Radii of the zones of inhibition are given in mm. “/” not tested.

## Supplementary Materials

The following are available online at www.mdpi.com/1660-3397/14/11/201/s1, Figures S1–S47: Spectra of the new compounds **1**–**7**.
