# Supplementary material for: Chemistry and Bioactivity of Briaranes from the South China Sea Gorgonian Dichotella gemmacea
Source: Mar Drugs. 2016 Oct 28;14(11):201. doi: 10.3390/md14110201 (PMC5128744; doi:10.3390/md14110201)
Supplement: Supplementary file 1 [file marinedrugs-14-00201-s001.docx]

Supplementary Materials: Chemistry and Bioactivity of Briaranes from the South China Sea Gorgonian *Dichotella gemmacea*

Cui Li, Ming-Ping La, Hua Tang, Peng Sun, Bao-Shu Liu, Chun-Lin Zhuang, Yang-Hua Yi and Wen Zhang

Index

| 1 | Spectra of the new compound **1** | S3–S8 |
| --- | --- | --- |
|  | HRESIMS spectrum of the new compound **1** | S3 |
|  | ^1^H NMR spectrum of the new compound **1** | S3 |
|  | ^13^C NMR spectrum of the new compound **1** | S4 |
|  | HSQC spectrum of the new compound **1** | S5 |
|  | ^1^H–^1^H COSY spectrum of the new compound **1** | S6 |
|  | HMBC spectrum of the new compound **1** | S7 |
|  | NOESY spectrum of the new compound **1** | S8 |
| 2 | Spectra of the new compound **2** | S9–S13 |
|  | HRESIMS spectrum of the new compound **2** | S9 |
|  | ^1^H NMR spectrum of the new compound **2** | S9 |
|  | ^13^C NMRspectrum of the new compound **2** | S10 |
|  | HSQC spectrum of the new compound **2** | S11 |
|  | HMBC spectrum of the new compound **2** | S12 |
|  | NOESY spectrum of the new compound **2** | S13 |
| 3 | Spectra of the new compound **3** | S14–S20 |
|  | HRESIMS spectrum of the new compound **3** | S14 |
|  | ^1^H NMR spectrum of the new compound **3** | S15 |
|  | ^13^C NMR spectrum of the new compound **3** | S16 |
|  | HSQC spectrum of the new compound **3** | S17 |
|  | ^1^H–^1^H COSY spectrum of the new compound **3** | S18 |
|  | HMBC spectrum of the new compound **3** | S19 |
|  | NOESY spectrum of the new compound **3** | S20 |
| 4 | Spectra of the new compound **4** | S20–S27 |
|  | HRESIMS spectrum of the new compound **4** | S20 |
|  | ^1^H NMR spectrum of the new compound **4** | S20 |
|  | ^13^C NMR spectrum of the new compound **4** | S21 |
|  | HSQC spectrum of the new compound **4** | S21 |
|  | ^1^H–^1^H COSY spectrum of the new compound **4** | S22 |
|  | HMBC spectrum of the new compound **4** | S23 |
|  | NOESY spectrum of the new compound **4** | S24 |
| 5 | Spectra of the new compound **5** | S25–S29 |
|  | HRESIMS spectrum of the new compound **5** | S25 |
|  | ^1^H NMR spectrum of the new compound **5** | S25 |
|  | ^13^C NMR spectrum of the new compound **5** | S26 |
|  | HSQC spectrum of the new compound **5** | S27 |
|  | ^1^H–^1^H COSY spectrum of the new compound **5** | S28 |
|  | HMBC spectrum of the new compound **5** | S29 |
| 6 | Spectra of the new compound **6** | S30–S45 |
|  | HRESIMS spectrum of the new compound **6** | S30 |
|  | ^1^H NMR spectrum of the new compound **6** | S30 |
|  | ^13^C NMR spectrum of the new compound **6** | S31 |
|  | HSQC spectrum of the new compound **6** | S32 |
|  | ^1^H-^1^H COSY spectrum of the new compound **6** | S33 |
|  | HMBC spectrum of the new compound **6** | S34 |
|  | NOESY spectrum of the new compound **6** | S45 |
| 7 | Spectra of the new compound **7** | S36–S41 |
|  | HRESIMS spectrum of the new compound **7** | S36 |
|  | ^1^H NMR spectrum of the new compound **7** | S36 |
|  | ^13^C NMR spectrum of the new compound **7** | S37 |
|  | HSQC spectrum of the new compound **7** | S38 |
|  | ^1^H-^1^H COSY spectrum of the new compound **7** | S39 |
|  | HMBC spectrum of the new compound **7** | S40 |
|  | NOESY spectrum of the new compound **7** | S41 |

**Figure S1.** HRESIMS spectrum of the new compound **1**.

**Figure S2.** ^1^H NMR (400 MHz, CDCl_3_) spectrum of the new compound **1**.

**Figure S3.** ^13^C NMR (100 MHz, CDCl_3_) spectrum of the new compound **1**.

**Figure S4.** HSQC spectrum of the new compound **1**.

**Figure S5.** ^1^H–^1^H COSY spectrum of the new compound **1**.

|  |
| --- |
| 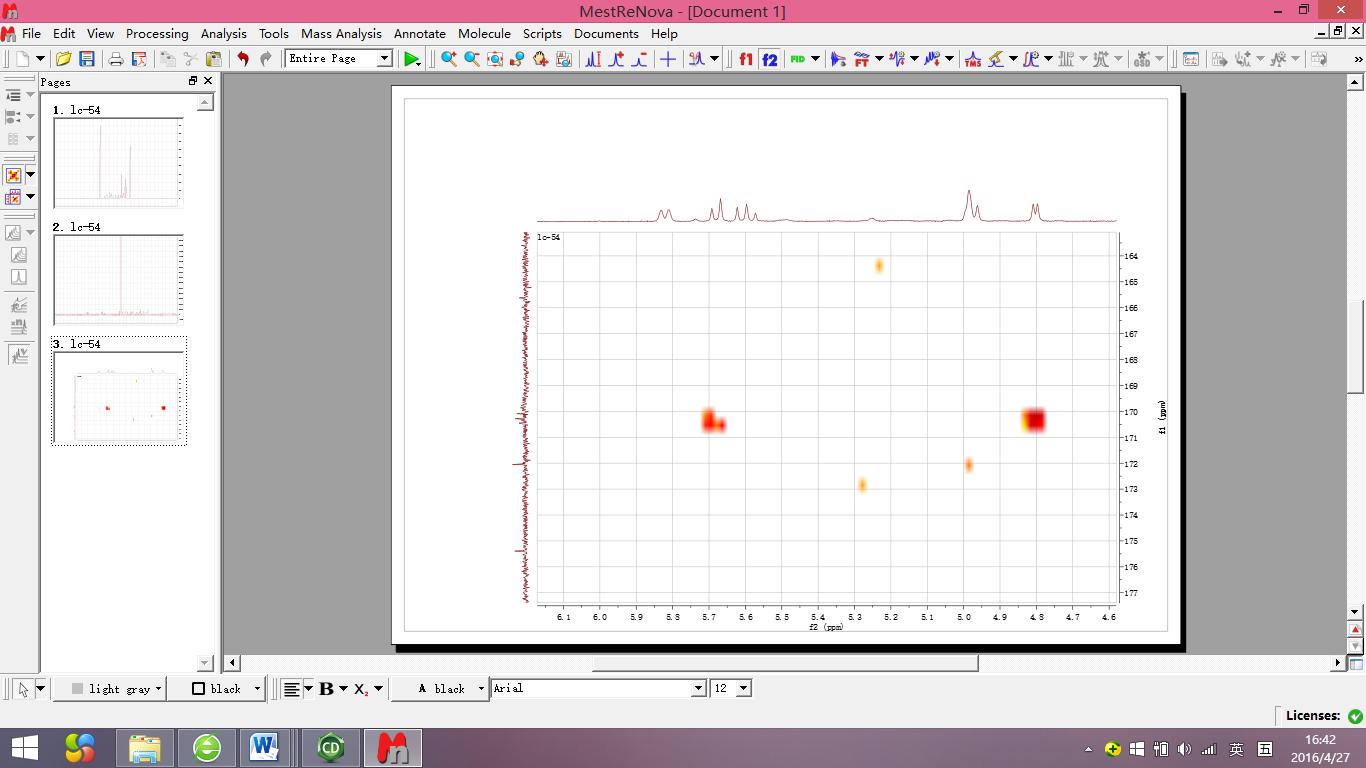 |

**Figure S6.** HMBC spectra of the new compound **1**.


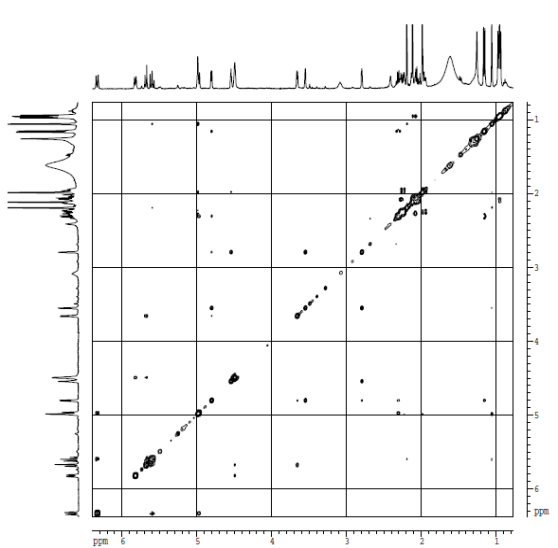


**Figure S7.** NOESY spectrum of the new compound **1**.

**Figure S8.** HRESIMS spectrum of the new compound **2**.

**Figure S9.** ^1^H NMR (400 MHz, CDCl_3_) spectrum of the new compound **2**.

**Figure S10.** ^13^C NMR (100 MHz, CDCl_3_) spectrum of the new compound **2**.

**Figure S11.** HSQC spectrum of the new compound **2**.

**Figure S12.** HMBC spectrum of the new compound **2**.

**Figure S13.** NOESY spectrum of the new compound **2**.

**Figure S14.** HRESIMS spectrum of the new compound **3**.

**Figure S15.** ^1^H NMR (400 MHz, CDCl_3_) spectrum of the new compound **3**.


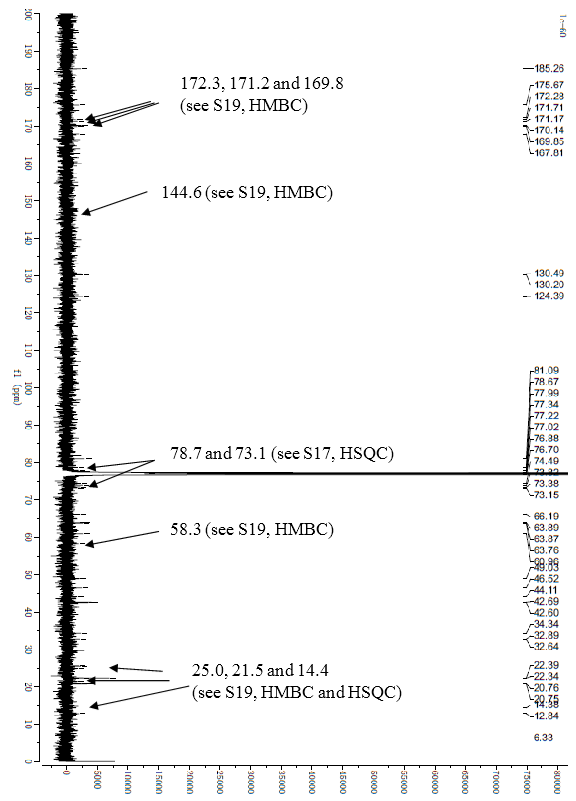


**Figure S16.** ^13^CNMR (100 MHz, CDCl_3_) spectrum of the new compound **3**.

| **** |
| --- |
| **** |

**Figure S17.** HSQC spectra of the new compound **3**.


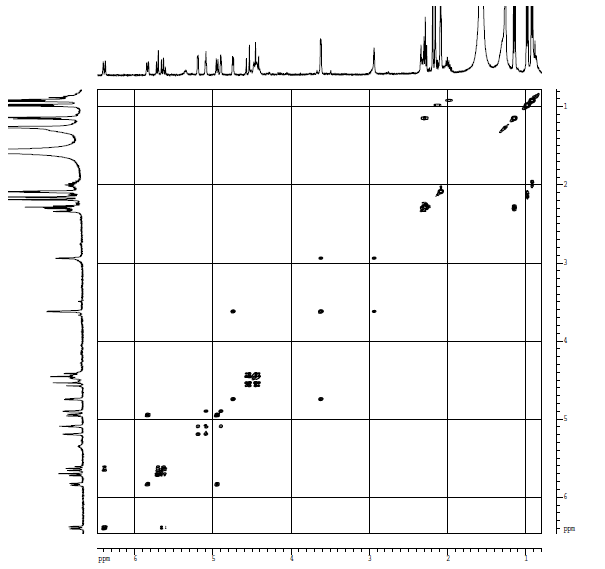


**Figure S18.** ^1^H–^1^H COSY spectrum of the new compound **3**.

|  |
| --- |
| **** |

**Figure S19.** HMBC spectra of the new compound **3**.

**Figure S20.** NOESY spectrum of the new compound **3**.

**Figure S21.** HRESIMS spectrum of the new compound **4**.

**Figure S22.** ^1^H NMR (400 MHz, CDCl_3_) spectrum of the new compound **4**.

**Figure S23.** ^13^C NMR (100 MHz, CDCl_3_) spectrum of the new compound **4**.

**Figure S24.** HSQC spectrum of the new compound **4**.

**Figure S25.** ^1^H–^1^H COSY spectrum of the new compound **4**.

**Figure S26.** HMBC spectrum of the new compound **4**.

**Figure S27.** NOESY spectrum of the new compound **4**.

**Figure S28.** HRESIMS spectrum of the new compound **5**.

**Figure S29.** ^1^HNMR (400 MHz, CDCl_3_) spectrum of the new compound **5**.

**Figure S30.** ^13^C NMR (100 MHz, CDCl_3_) spectrum of the new compound **5**.

**Figure S31.** HSQC spectrum of the new compound **5**.

**Figure S32.** ^1^H–^1^H COSY spectrum of the new compound **5**.

**Figure S33.** HMBC spectrum of the new compound **5**.

**Figure S34.** HRESIMS spectrum of the new compound **6**.

**Figure S35.** ^1^H NMR (400 MHz, CDCl_3_) spectrum of the new compound **6**.


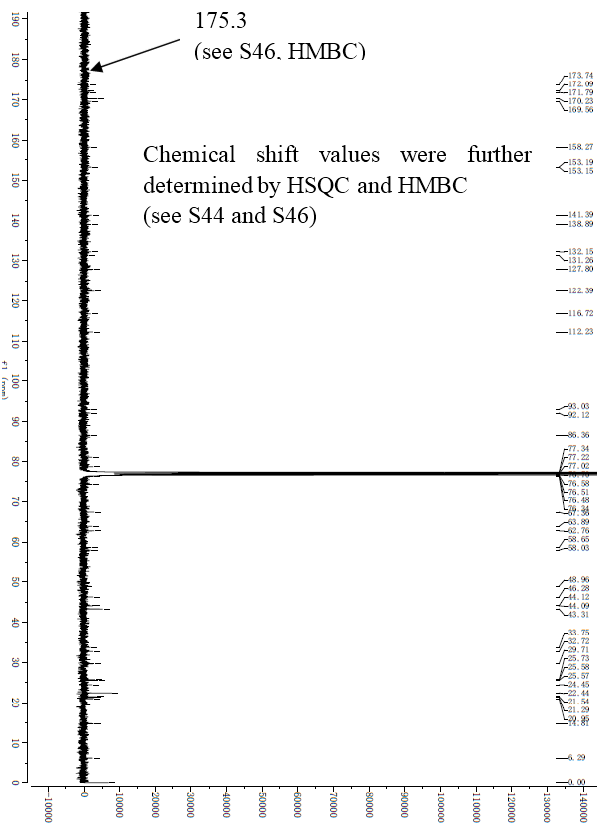


**Figure S36.** ^13^C NMR (100 MHz, CDCl_3_) spectrum of the new compound **6**.

|  |
| --- |
| **** |

**Figure S37.** HSQC spectra of the new compound **6**.

**Figure S38.** ^1^H–^1^H COSY spectrum of the new compound **6**.

**Figure S39.** HMBC spectrum of the new compound **6**.

**Figure S40.** NOESY spectrum of the new compound **6**.

**Figure S41.** HRESIMS spectrum of the new compound **7**.

**Figure S42.** ^1^H NMR (400 MHz, CDCl_3_) spectrum of the new compound **7**.

**Figure S43.** ^13^C NMR (100 MHz, CDCl_3_) spectrum of the new compound **7**.

**Figure S44.** HSQC spectrum of the new compound **7**.

**Figure S45.** ^1^H–^1^H COSY spectrum of the new compound **7**.

|  |
| --- |
|  |

**Figure S46.** HMBC spectra of the new compound **7**.

**Figure S47.** NOESY spectrum of the new compound **7**.
